# A spatiotemporal data mining study to identify high-risk neighborhoods for out-of-hospital cardiac arrest (OHCA) incidents

**DOI:** 10.1038/s41598-022-07442-7

**Published:** 2022-03-03

**Authors:** Paulina Pui-yun Wong, Chien-Tat Low, Wenhui Cai, Kelvin Tak-yiu Leung, Poh-Chin Lai

**Affiliations:** 1 Science Unit, Lingnan University, Tuen Mun, Hong Kong SAR; 2 Institute of Policy Studies, Lingnan University, Tuen Mun, Hong Kong SAR; 3 LEO Dr David P. Chan Institute of Data Science, Lingnan University, Tuen Mun, Hong Kong SAR; 4 Department of Geography, The University of Hong Kong, Pok Fu Lam, Hong Kong SAR; 5 Ambulance Service Institute (Hong Kong), Hong Kong, Hong Kong SAR

**Keywords:** Cardiovascular diseases, Mathematics and computing

## Abstract

Out-of-hospital cardiac arrest (OHCA) is a worldwide health problem. The aim of the study is to utilize the territorial-wide OHCA data of Hong Kong in 2012–2015 to examine its spatiotemporal pattern and high-risk neighborhoods. Three techniques for spatiotemporal data mining (SaTScan’s spatial scan statistic, Local Moran’s I, and Getis Ord Gi*) were used to extract high-risk neighborhoods of OHCA occurrence and identify local clusters/hotspots. By capitalizing on the strengths of these methods, the results were then triangulated to reveal “truly” high-risk OHCA clusters. The final clusters for all ages and the elderly 65+ groups exhibited relatively similar patterns. All ages groups were mainly distributed in the urbanized neighborhoods throughout Kowloon. More diverse distribution primarily in less accessible areas was observed among the elderly group. All outcomes were further converted into an index for easy interpretation by the general public. Noticing the spatial mismatches between hospitals and ambulance depots (representing supplies) and high-risk neighborhoods (representing demands), this setback should be addressed along with public education and strategic ambulance deployment plan to shorten response time and improve OHCA survival rate. This study offers policymakers and EMS providers essential spatial evidence to assist with emergency healthcare planning and informed decision-making.

## Introduction

Out-of-hospital cardiac arrest (OHCA), a condition of sudden collapse due to cardiac disorder, is a common medical issue and a major contributor to global mortality^1^. The OHCA survival rate of 1.25% in Hong Kong^2^ is among the lowest in Asia^3,4^. Many factors apart from personal characteristics—including environmental, sociodemographic, and geographical influences—can put a person at risk of sudden cardiac arrest. A local study reported that OHCA in Hong Kong showed greater impact on males, the elderly 65+, overweight individuals, and people suffering from cardiovascular and respiratory issues^5^. Also, daily temperature was strongly related to OHCA incidence in Hong Kong, whereby a unit decrease in temperature (°C) would result in a 5.6% increase in OHCA cases^5^.

The emergency medical services (EMS) in Hong Kong is a one-tier system provided by the Hong Kong Fire Services Department (FSD) aided by other auxiliary/voluntary ambulance services. OHCA resuscitation mainly includes providing basic life support and defibrillation using an automated external defibrillator (AED), along with intravenous fluids and airway management via a laryngeal mask^6,7^. The FSD target is to meet response time in 92.5% of all EMS calls within 12 min (from the time of call to the arrival of an ambulance at the street address). A study based on data in 2012–2013^2^ reported that the median response time was about 9 min, shorter than the EMS targeted time, which was also in the mid-range among Asian cities^4,8^. However, the median time of 12 min for call-to-first defibrillation time in the same period was deemed too long, compared with other developed countries of comparable density and accessibility of AED^6^. The bystander AED rate in the same period was also found to be very low (1.4%) as a result of inadequate public knowledge^9,10^. Hong Kong has over 5000 OHCA cases annually, most of which occurred in the home environment^2^. More than half of the residents do not have first aid training and only 18% can use an AED^9,10^. Thus, we can summarize that the key modifiable factors to improving OHCA survival in Hong Kong are (i) faster ambulance response time and shorter distance to Accident and Emergency (A&E) services, (ii) widespread availability of AED, and (iii) enhanced knowledge and ability of bystanders to offer first-aid. Realizing that it would be difficult if not impossible to improve on response time due to worsening traffic congestion in Hong Kong^6^, a practical approach to increasing service efficiency would involve identifying high-risk OHCA hotspots and strategically increasing the number of ambulance depots in these neighborhoods. There is also the need to raise public awareness and knowledge about cardiopulmonary resuscitation (CPR)^11,12^.

The primary goal of this study is to identify OHCA clusters (areas with high OHCA incidents) and high-risk neighborhoods in Hong Kong. Various spatiotemporal data mining/statistical techniques, also known as spatial cluster analyses, were employed in this study: SaTScan’s spatial scan statistic^13^, Local Moran’s I^14^, and Getis Ord Gi*^15^. High risk OHCA neighborhoods are defined as local clusters or hotspots, where the frequency of OHCA occurrence is higher than expected. To derive OHCA clusters, the Getis-Ord Gi* makes use of a self-defined threshold distance instead of the inverse distance measure employed by Moran’s I or the statistical scanning technique used by SaTScan. We note that different spatiotemporal data mining approach would yield different patterns of OHCA clusters. Without a consensus on the best clustering method and considering the uneven landscape of Hong Kong characterized by hilly lands interspersed with waterbodies, the combined use of three methods would capitalize on individual strengths and allow impartial identification of high-risk neighborhoods. Previous literature have also recommended such an approach that has become common practice to integrate different methods into one new index so as to improve the results and produce optimum output^16,17^.

The spatiotemporal data mining of all population was stratified by age (all ages vs elderly 65+) and calendar year (2012 to 2015 inclusive). The outcomes by the three clustering methods were subsequently compared and triangulated in order to identify “credible” high-risk OHCA clusters, i.e. those recognized by at least two analytical methods as potential sites for community-based improvements. These “credible” high-risk neighborhoods for all ages and the elderly were further displayed as risk maps to visually evaluate accessibility and sufficiency of medical facilities. Identification of OHCA clusters will assist the Government and EMS providers in better appreciating any misfits in healthcare planning and resource allocation, which is particularly relevant to Hong Kong with rapidly ageing population.

## Methods

### Study area

**Figure 1:**
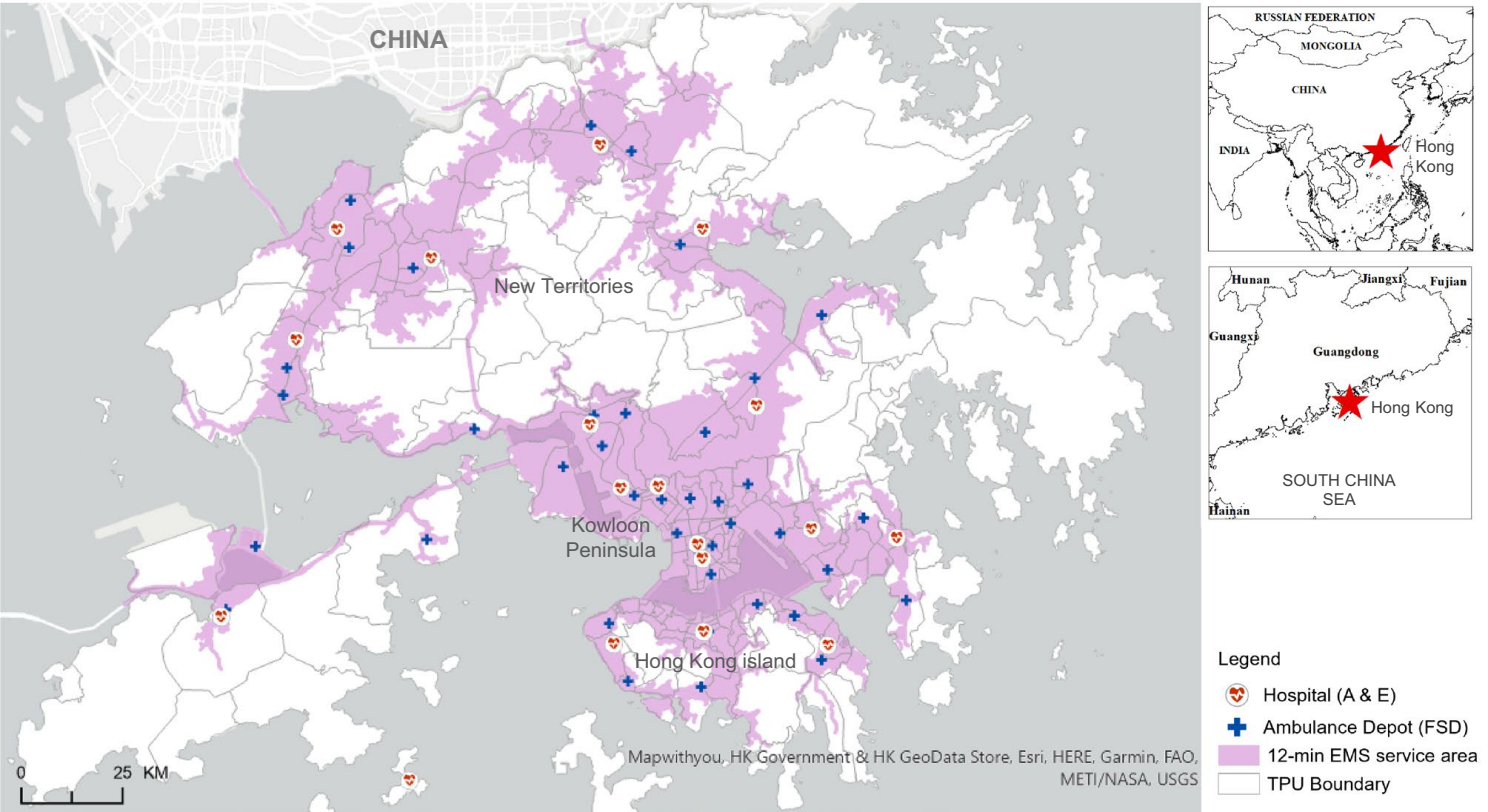
A map of the Hong Kong Special Administrative Region of China. The area is partitioned by tertiary planning units (TPUs) and emergency medical service (EMS) areas with targeted 12-min response time (shaded in pink). Areas not shaded represent country parks or non-populated lands. The map also shows locations of ambulance depots managed by the Fire Services Department (FSD) and hospitals with accident and emergency (A&E) services. (Generated by ArcGIS 10.7, URL: http://www.esri.com/software/arcgis/arcgis-for-desktop).

The Hong Kong Special Administrative Region (SAR) of China (Fig. 1) is situated at longitude 114° 15′ N and latitude 22° 15′ E, covering a total land area of 1106.66 km^2^. Hong Kong is one of the most densely populated megacities in the world with a high density of tall buildings and skyscrapers. Its 2020 total population was approximately 7.5 million, which was equivalent to 6,890 persons per km^2^ (Census and Statistics Department of Hong Kong, https://www.censtatd.gov.hk/). Its typical subtropical hot and humid weather, along with compact urban living with limited open space, has resulted in severe thermal discomfort and high energy consumption of the city. Figure 1 illustrates a map of Hong Kong, with administrative boundaries of 209 tertiary planning units (TPUs), locations of ambulance depots and hospitals, and designated service areas for the targeted EMS response time of 12 min^10,18^. A TPU is an administrative unit used by the Hong Kong Special Administrative Region (HKSAR) for town planning purposes. It is demarcated and revised by the HKSAR Planning Department every census year for the compilation of population census.

### Data acquisition and pre-processing

A total of 19,658 OHCA cases occurred in Hong Kong between January 2012 and December 2015, according to official data collected and managed by the EMS of FSD. All cases were extracted from the Hong Kong emergency call database/dispatch systems and were reported according to the Utstein style^2,19^. Incomplete and duplicated records have been excluded and cleaned by the EMS^5^. Each OHCA record was anonymized and contained demographic data (i.e. age, gender, medical condition) with relevant information from the incident report (i.e. date, time, location of cardiac arrest, treatment rendered, outcome). The study employed ArcMap 10.7 for processing these data for spatial analyses and map visualization.

The OHCA cases were geocoded into *x,y* coordinate pairs (in HK 1980 Grid System) and then aggregated by TPUs, to protect individual privacy, for statistical analysis. All OHCA cases covering every types of location of cardiac arrest were first aggregated by TPUs for year 2011 and further stratified by calendar year (2012–2015) and age group (all ages vs. elderly 65+). The OHCA cases for all ages and elderly 65+ were weighted by total population and total elderly population of the corresponding TPUs respectively. The locations of ambulance depots and A&E hospitals shown in Fig. 1 were obtained from the Hong Kong open data portal (Office of the Government Chief Information Officer, https://data.gov.hk/).

### Spatial analysis and statistics

**Figure 2:**
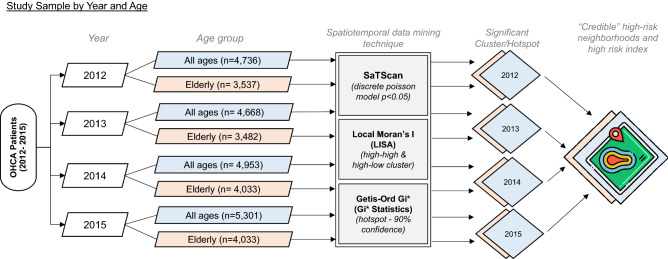
A diagrammatic representation of the spatiotemporal data mining techniques used in analyzing 19,658 OHCA cases in the study.

Spatial and spatiotemporal statistics are widely used in disease surveillance to identify geographic areas of elevated disease risk and for early detection of disease outbreaks^13^ (SaTScan: version 8.0. Albany, NY, USA, 2018). The study employed SaTScan’s spatial scan statistic, Local Moran’s I, and Getis Ord Gi* to identify high-risk OHCA clusters. Figure 2 shows that each analysis method was computed separately for 2012–2015 calendar year for all ages cases (boxes shaded in cyan). With a substantial 75.25% of the OHCA cases belonging to the elderly population, a subset of cluster analyses was also computed for this age group (boxes shaded in pink). The results of statistical cluster analyses with hotspots were spatially overlaid and triangulated to yield “credible” high-risk OCHA neighborhoods separately for all ages and elderly groups. These results can be visualized and displayed in map form.

#### SaTScan’s spatial scan statistics

SaTScan™ is a free software widely employed for analyzing spatial and temporal data for detecting disease clusters, such as HIV^20^, Legionnaires’ disease^21^, OHCA cases^22^ and tick-borne disease in the U.S.^23^ The study applied a discrete Poisson-based model in SaTScan to examine OHCA cases in circular areas of varying diameters across Hong Kong^20^, assuming that the number of cases in a geographical location follows the Poisson distribution based on a known underlying population at risk^24^. Relative risks were calculated to reveal the risk level of each OHCA cluster. A spatial cluster with a high concentration of point locations would be regarded as a high-risk OHCA area^20^. Monte Carlo simulation using 1000 replications was used to detect statistically significant (*p* < 0.05) clusters^22,25^. The Gini coefficient was also calculated for each set of clusters to obtain a more refined collection of non-overlapping clusters^26^. These “Gini clusters” were ranked by statistical significance and each was assigned a numerical score. The OHCA clusters defined in this manner were then visualized using ArcMap 10.7.1 for subsequent spatial analysis and triangulation.

#### Local Moran’s I statistic

Local Moran’s I, also known as Local Indicators of Spatial Association (LISA), measures similarity among areas and defines neighborhoods with similar values as a cluster^14^. It also identifies dissimilar neighborhoods or spatial outliers. This study used ArcMap 10.7.1 to compute Local Moran’s I by considering the spatial distribution of OHCA cases and isolating areas with significantly higher or lower number of clusters than expected. The approach applied the inverse distance weights in establishing spatial relationships for the clustering analysis^14^.

The Moran’s I index lies within the [− 1, 1] range to denote negative/positive spatial clustering effects with the zero value denoting a random distribution. Using a 95% confidence level, a statistically significant and positive z-score implies that the surrounding neighborhoods share similar OHCA rates. An area surrounded by neighborhoods with similar high z-score values yields a “High-High” (HH) cluster (i.e., a hotspot). Conversely, an area surrounded by neighborhoods with similar low z-scores is regarded as a “Low-Low” (LL) cluster (i.e., a coldspot). In this study, neighborhoods with HH and HL clusters were determined as high-risk OHCA clusters/neighborhoods.

#### Getis-Ord Gi* statistic

The Getis-Ord Gi* statistic (Gi* statistic) measures the degree of spatial clustering with statistical significance at different spatial scales^15,16^. Using ArcMap 10.7.1, the Gi* calculation produces z-scores (GiZScore) and associated p-values (GiPValue) to indicate statistical significance of a particular neighborhood as a part of spatial clusters of either high- or low-values. A larger z-score denotes more intense clustering of higher OCHA incidents (i.e., a hotspot) whereas a smaller z-score signifies more intense clustering of low OCHA incidents (i.e., a coldspot). Neighborhoods in which hotspots were identified at 90% confidence were selected as high-risk OHCA clusters/neighborhoods.

### High-risk index

A high-risk OCHA neighborhood/TPU was denoted as “credible” if the TPU was rated high-risk by at least two of the three spatiotemporal clustering techniques described above (see also Fig. 2). The spatial clusters for all ages and the elderly groups were processed separately for each calendar year and triangulated using spatial overlay in ArcMap. TPUs rated as high-risk by all three techniques for a given year were denoted as Level 1 TPUs and those rated as high-risk by two of the three techniques were labeled as Level 2 TPUs.

To facilitate easy interpretation by the general public, Level 1 and Level 2 TPUs were assigned 1 and 0.5 scores respectively. By performing spatial overlay of the clustering results of all four years (2012–2015), the total score for a “credible” high-risk neighborhood/TPU would range between 0.5 and 4, hereafter referred to as the high-risk index of OHCA occurrence. The index was further translated into four ordinal classes (0.5–1.0 = low, 1.5–2.0 = medium, 2.5–3.0 = high, and 3.5–4 = extremely high). A high-risk index of “extremely high” (i.e. TPUs identified as high-risk by all three clustering methods in each of the four years) thus denotes “credible” high-risk neighborhoods with a more urgent need for service upgrade. Similarly, a high-risk index of “low” (i.e. TPUs rated as high-risk by two of three clustering methods during the four-year study period) denotes “credible” high-risk neighborhoods of lower priority for service improvement according to available resources.

## Results

Table 1 summarizes characteristics of the 19,658 OHCA cases distributed in 209 TPUs in 2012–2015. The mean age of OHCA patients was 75.67 and more than three-quarters was elderly 65+. Males accounted for 55.22% and females for 44.32% of the sample. The majority of OHCA events was not related to trauma (n = 18,747; 95.37%). The “No ROSC” rate showing without return of spontaneous circulation was high (n = 18,269; 92.93%), with the majority of these events occurring at home (n = 10,383; 52.82%). Nearly 30% (n = 5851) of the OHCA cases resided in a home for the aged (HFA) or a nursing home for the elderly.

**Table 1:** Characteristics and descriptive statistics of the study sample (n = 19,658).

	2012	2013	2014	2015	n	(%)
**General**						
All ages OHCA cases	4736	4668	4953	5301	19,658	100.00
Elderly 65+ OHCA cases	3537	3482	3741	4033	14,793	75.25
Total population*	7,070,388					
Total elderly 65+ population*	941,205					
**Age**						
Mean	75.36	75.46	75.99	75.84	–	–
Maximum	108	112	108	111	–	–
Minimum	0	0	0	0	–	–
Standard Deviation	17.35	17.51	17.74	17.84	–	–
**Type of OHCA**						
Trauma	105	86	85	113	389	1.98
Non-Trauma	4448	4451	4778	5070	18,747	95.37
Unknown	183	131	90	118	522	2.66
**Gender**						
Female	2089	2027	2210	2386	8712	44.32
Male	2622	2620	2719	2894	10,855	55.22
Unknown	25	21	24	21	91	0.46
**ROSC**						
At A&E	299	224	151	187	861	4.38
Pre-Hosp	122	127	105	133	487	2.48
No ROSC	4296	4314	4693	4966	18,269	92.93
Unknown	19	3	4	15	41	0.21
**Location of cardiac arrest**						
En-route to Hospital	154	170	167	193	684	3.48
HFA	1468	1391	1482	1510	5851	29.76
Home	2440	2439	2653	2851	10,383	52.82
Public Place	422	406	391	453	1672	8.51
Street	150	151	155	185	641	3.26
Unknown	102	111	105	109	427	2.17

*ROSC* Return of spontaneous circulation, *HFA* Home for the aged.

*Based on 2011 population census.

### Analysis of findings by spatial clustering methods irrespective of age

**Figure 3:**
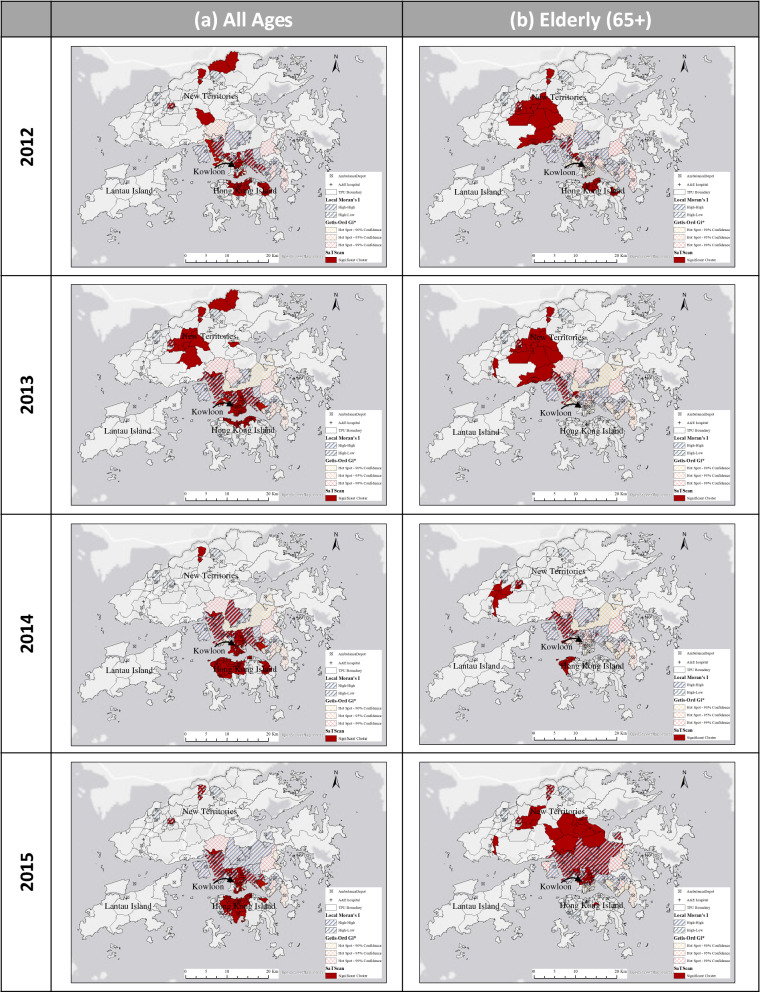
Distribution of high-risk neighborhoods using three spatial clustering techniques (SaTScan, Local Moran’s I, and Getis Ord Gi*). (**a**) Results for all ages based on 19,658 OHCA cases in 2012–2015. (**b**) Results for the elderly (65+) group based on 14,793 OHCA cases in 2012–2015. (Generated by ArcGIS 10.7, URL: http://www.esri.com/software/arcgis/arcgis-for-desktop).

Table 2 summarizes the number of high-risk neighborhoods/TPUs detected by the three spatial clustering methods. It also shows the number of Level 1 and Level 2 “credible” high-risk neighborhoods. The results show differing numbers of spatial clusters were identified by different methods. These spatial clusters were mapped to examine the degree of agreement or discrepancy in terms of spatial locations. Figure 3 shows results of the spatial overlay by three clustering methods for all ages (column a) and elderly 65+ (column b) for each year in the study period. Significant clusters computed by SaTScan were shaded in maroon; the HH (high clusters neighbored by high clusters) and HL (high clusters neighbored by low clusters) clusters produced by Local Moran’s I were represented using gray slanting lines; and outcomes from Getis Ord Gi* (hotspots identified at 90% confidence) were displayed in orange–red crossed pattern.

**Table 2:** Number of high-risk neighborhoods by spatial clustering methods (SaTScan, Local Moran’s I, and Getis Ord Gi*).

Method	All ages				Elderly (65+)			
	2012	2013	2014	2015	2012	2013	2014	2015
**SaTScan**								
Significant Cluster (Gini)	12	6	7	5	7	5	7	4
**Local Moran’s I (LISA)**								
High-high	38	33	35	40	31	38	42	68
High-low	4	3	4	8	4	5	4	8
Getis Ord Gi*								
90% significance level	14	7	12	8	11	11	13	9
95% significance level	34	24	18	23	37	34	20	30
99% significance level	26	27	41	43	29	36	46	84
**Triangulated**								
Level 1								
(SaTScan, LISA & Gi*)	16	22	21	22	6	5	7	29
Level 2								
(SaTScan & LISA)	16	22	21	22	6	5	7	29
(SaTScan & Gi*)	17	26	24	22	2	2	1	20
(LISA & GI*)	22	11	14	18	31	31	29	11

Level 1 denotes “credible” high-risk neighborhoods identified by all three clustering methods; Level 2 denotes “credible” high-risk neighborhoods identified by two of three clustering methods.

**Figure 4:**
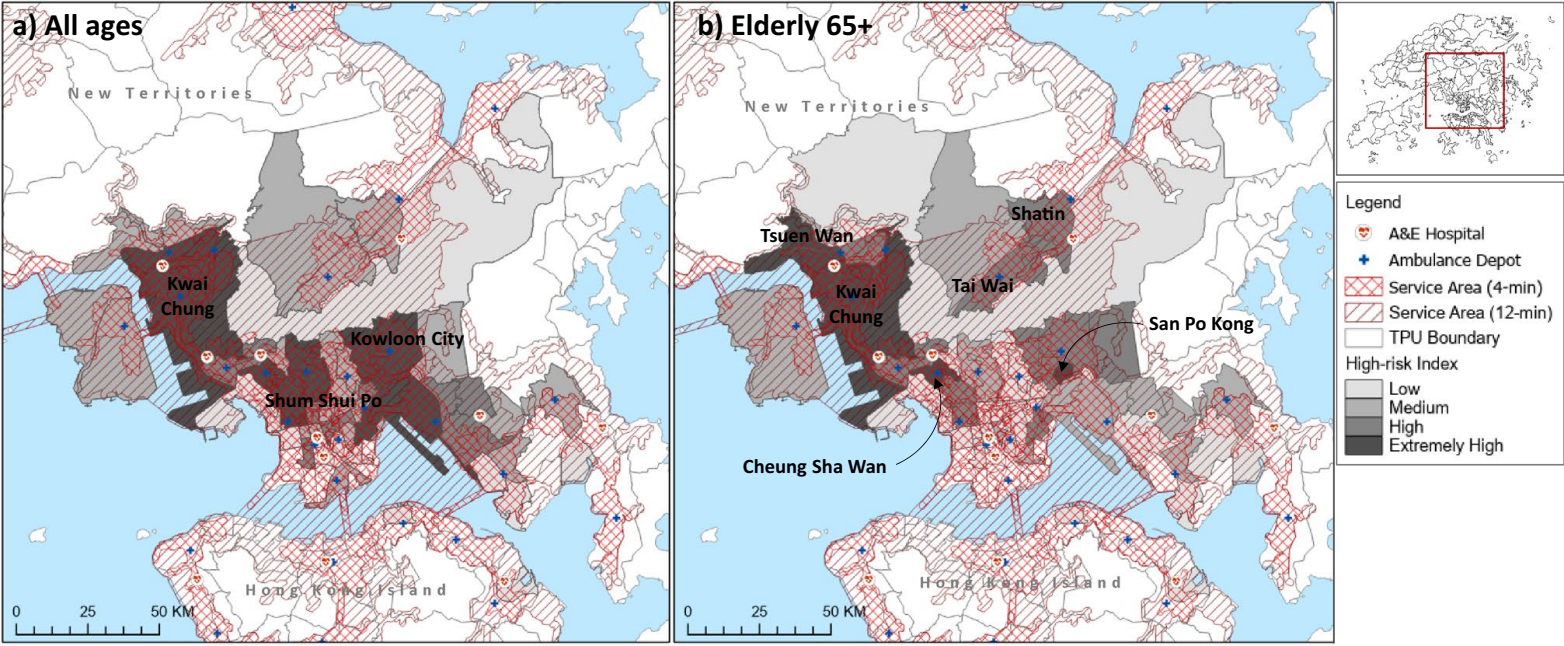
Results based on high-risk indices for all ages and elderly 65+. The 4-min ideal and 12-min targeted response areas (from hospitals with A&E departments and ambulance depots) are shown to draw attention to high-risk neighborhoods with inadequate access to emergency medical services. (Generated by ArcGIS 10.7, URL: http://www.esri.com/software/arcgis/arcgis-for-desktop).

Referring to Table 2 and Fig. 3, the spatial distribution of high-risk clusters for all ages by different clustering methods appeared to have some agreement as evidenced by the spatial overlap. It was observed that “credible” high-risk neighborhoods for each year tended to concentrate in the Kowloon area (including districts of Yau Tsim Mong, Sham Shui Po, and Kowloon City) with statistically significant high-risk clusters concentrating in Kwai Chung, Kwun Tong, and Kowloon East (refer to Fig. 4 for geographic positions). These places are not only fast paced and densely populated but also local economic and political centers. There exist older neighborhoods with poor road infrastructure and severe traffic congestion from intensified urban development that collectively account for the increase in the overall ambulance response time within the region.

### Analysis of findings by three spatial clustering methods for elderly 65+

The distribution of high-risk clusters for the elderly group by different clustering methods yielded quite different results, compared with all ages group, both in terms of the number of high-risk clusters (Table 2) and the spatial locations (Fig. 3). Close examination of the spatial distribution of OHCA clusters for elderly 65+ revealed more dispersed distribution in 2014, compared with earlier years, whereby significant clusters were found in the New Territories covering suburban and rural areas. Almost similar patterns were observed in 2015 with the exception of significant clusters appearing in the Kowloon region. The suburban and rural areas were not within the spatial coverage of 12-min ambulance service area targeted by the FSD (see Fig. 1).

Several “credible” high-risk clusters were detected for elderly 65+ but the number was much smaller compared with that of the full sample. These significant and high-risk clusters were concentrated in Kwai Ching, Tsuen Wan, Yuen Long, and rural villages. The locations of these high-risk OHCA clusters suggest that elderly 65+ individuals tended to reside in older neighborhoods with narrow streets (e.g., Kwai Ching) and less accessible rural areas. The longer EMS response time thus increased the risk of adverse OHCA outcomes.

### Analysis of findings based on high-risk index

Figure 4 illustrates results of the high-risk indices for all ages and elderly 65+. The darker shading denotes “credible” high-risk neighborhoods with “extremely high” likelihood of OHCA occurrence. These neighborhoods should receive priority attention to service upgrade if EMS was deemed insufficient. The lighter shading shows “credible” high-risk neighborhoods with lower risks of OHCA occurrence. These neighborhoods are of lower priority for consideration of EMS service upgrade and when resources become available.

The locations of A&E departments and ambulance depots were also displayed in Fig. 4 to reveal current levels of service provision within the 4-min ideal and 12-min targeted spatial coverage. The spatial distribution of A&E departments and ambulance depots is geographically uneven, which is particularly problematic for people living in high-risk neighborhoods. “Credible” high-risk neighborhoods/TPUs including San Po Kong, Kowloon City, and Shum Shui Po lack EMS within the vicinity of 4-min ideal response time for OHCA^18^.

## Discussion

Drawing on the strengths of individual spatial clustering methods and integrating the three sets of clustering results, “credible” high-risk neighborhoods can be determined with a high degree of confidence. The study also reveals that the SaTScan and Local Moran’s I approaches yielded highly consistent results even for Hong Kong, a metropolitan area known for its high spatial diversity of hilly and interrupted urban landscapes. This integrative approach can be adapted to examine community-based problems and facility distribution in other cities.

The study also shows the need to examine specific population group, such as elderly 65+, to detect spatial variation of hotspots from the general population. Our findings reported that the OHCA hotspots for elderly 65+ were distributed mostly in older neighborhoods and the less accessible suburban and rural areas where more affordable nursing homes were located^27,28^. This is particularly problematic as first aid assistance for OHCA should be provided within 4 min^18^ to increase the survival rate of patients. There is thus a need to improve EMS provision for elderly 65+. Besides strategically increasing the number of EMS facilities^29^ in high-risk neighborhoods, other community-based interventions such as improvement of public CPR awareness and education^30^ may ultimately improve the survival rate of OHCA, as proven in previous litereature^31^.

This study has several limitations. First, the anonymized OHCA records provided by EMS did not include any personal risk factors (i.e. daily activity, socioeconomic status, occupation, etc.) and health condition or medical history before the cardiac arrest that would have influenced the association between the location of arrest and patient outcome. Second, prior treatment records of OHCA patients were not accessible to paramedics whilst en-route to hospitals or upon arrival at hospitals to enable effective treatment that could improve OHCA survival rate. Third, there is no official and centralized database about locations and conditions of AED in Hong Kong to enable further analyses on the density and accessibility of AED against the locations of arrest and OHCA occurrence.

## Conclusions

The paper illustrates three techniques for spatiotemporal data mining to identify high-risk OHCA neighborhoods using 2012–2015 data for Hong Kong. It describes how the techniques were integrated to derive a high-risk index for all ages and elderly 65+ population. The “credible” high-risk neighborhoods thus obtained can be used to assess the sufficiency level of EMS, i.e. the spatial coverage and adequacy of hospitals with A&E departments and ambulance depots in Hong Kong neighborhoods. Compared with relying on clustering results based on a single technique, this integrated approach that combines results from multiple techniques gives greater confidence in the generalizability of results. Moreover, examining different population groups can draw similarities and differences to better inform priorities to enable strategic planning in optimizing the location and number of EMS facilities^29^, as well as supplementing public CPR awareness and education, to improve the OHCA survival rate. The paper also suggests directions for future research by recognizing the limitations of the current study.

## Data Availability

The datasets used in the study are not publicly available and belong to the Hong Kong Fire Services Department. Request for use of data may be directed to the corresponding author.
